# Editorial: Peak frequencies in neural oscillatory activity and their connection to perception and cognition

**DOI:** 10.3389/fpsyg.2023.1234955

**Published:** 2023-06-23

**Authors:** Thomas J. Baumgarten, Andreas Wutz, Jason Samaha

**Affiliations:** ^1^Institute of Clinical Neuroscience and Medical Psychology, Medical Faculty, Heinrich Heine University Düsseldorf, Düsseldorf, Germany; ^2^Department of Psychology and Centre for Cognitive Neuroscience, University of Salzburg, Salzburg, Austria; ^3^Psychology Department, University of California, Santa Cruz, Santa Cruz, CA, United States

**Keywords:** neural oscillatory activity, neural oscillation, peak frequency, perception, cognition

Neural oscillatory activity has emerged as a central Research Topic in neuroscience (Buzsáki and Draguhn, 2004; Singer, 2018) from its early roots of scientific investigation almost a century ago (Berger, 1929; Adrian and Matthews, 1934). Since then, neural oscillations have been linked to a plethora of functions, including perception (Keil and Senkowski, 2018) and cognition (Ward, 2003), neural entrainment to internal- (Tort et al., 2018) or external (Schroeder and Lakatos, 2009) sensory inputs, neural information processing (Salinas and Sejnowski, 2001), and neural network synchronization (Jensen and Mazaheri, 2010; Fries, 2015). In parallel, the causal (Herrmann et al., 2016) or epiphenomenal (Ding and Simon, 2013) nature of neural oscillatory activity is a matter of continuous debate (Engel and Gerloff, 2022).

Regarding its parameterization, most studies focused on oscillatory power (i.e., the squared amplitude of a band-pass filtered signal) and phase (i.e., the current position within the oscillatory cycle). Despite their conceptually clear-cut operationalization, analyses of these parameters are confronted with an inherent problem: For which frequency range should these parameters be calculated and how should this range be determined? Initially, a priori-defined frequency ranges centered on a prominent power peak were linked to behaviorally relevant functions (Buzsáki and Draguhn, 2004). Importantly, however, this approach remains naïve to many critical aspects (Donoghue et al., 2022). First, it blurs inter-individual frequency differences (Haegens et al., 2014). Second, it can miss functionally important signal contributions if they lie outside of the predefined frequency boundaries (e.g., Lansbergen et al., 2011). Third, it potentially fails to assess if the signal really exhibits a spectral peak in the defined frequency range above and beyond the usual 1/f slope of aperiodic neural activity (He, 2014), resulting in the false parametrization of non-oscillatory background noise (Donoghue et al., 2020; Gerster et al., 2022). Together, these examples demonstrate the potential pitfalls of analyses based on a priori defined frequency bands, which highlights the importance of an empirically informed process of frequency band selection.

Peak frequency (PF; i.e., a signal's power-dominant frequency), has the potential to overcome the above-mentioned limitations. Computation of PF allows definition of recording-specific frequency peaks based on the empirical data, which can be used to compare individuals and/or experimental conditions. Additionally, when paired with novel tools correcting for aperiodic signal components (Wen and Liu, 2016; Donoghue et al., 2020), PF analyses reveal prominent frequency peaks in the signal. Thus, PF analysis allows for a more dynamic and refined view on the role of neural oscillatory activity.

In addition, PF itself may be a relevant variable for linking perceptual and cognitive functions to physiology (e.g., Pfurtscheller and Aranibar, 1977) and the scientific interest in PF has only increased in recent years (e.g., Michalareas et al., 2016; Xie et al., 2020). In this regard, two research avenues have been especially influential. First, the connection between parieto-occipital alpha-band PF and the temporal resolution of perception has gained major attention (Cecere et al., 2015; Samaha and Postle, 2015; Ronconi and Melcher, 2017). The core idea here is that individual PF drives the temporal sampling speed of incoming sensory information, with most evidence found for the visual domain. Second, multiple studies have highlighted the changes in PF across different neuropsychiatric diseases and its connection to symptom severity (e.g., Edgar et al., 2019; Ramsay et al., 2021). Here, a prominent aim is to establish PF changes as a biomarker for disease-related impairments.

The present Research Topic aims to promote PF as an important parameter in the study of neural oscillatory activity, its connection to perception and cognition, and its systematic alteration in neuropsychiatric diseases.

Two of the four contributions to this Research Topic examine the link between individual alpha PF and visual processing. Coldea et al. use EEG recordings and causal manipulation of alpha-band PF via TMS to differentiate subjective perceptual experience from objective task performance in a visual discrimination task. Whereas there was no influence of fixed 10 Hz TMS-manipulation before stimulus presentation, the EEG measures of individual alpha PF were linked to task performance and to its reaction to TMS. This study nicely demonstrates how PF recordings can inform analyses based on fixed, a priori vs. individually defined frequency ranges. Morrow et al. address the role of alpha-band PF for perceptual sampling by contrasting two competing hypotheses: rhythmic sampling (i.e., alpha oscillations phasically inhibit perceptual processing) and discrete perception (i.e., alpha oscillations temporally parse neuronal processing of perceptual input). Correlation analyses revealed no systematic connection between individual alpha PF and the latency of neural markers of visual processing. These findings indicate that individual alpha PF does not systematically shift early sensory processing in time.

Two other contributions studied the link between PFs and trait variables related to neuropsychiatric disorders. Strang et al. assessed if individual alpha-band PFs over occipitoparietal (i.e., classical alpha rhythm) and sensorimotor (i.e., mu rhythm) areas are linked to autism spectrum disorders. EEG recordings of neurotypical participants during motor execution and movement observation revealed that individuals with slower sensorimotor PFs exhibited higher autism-spectrum traits. This finding adds important information about potential biomarkers, since research so far largely focused on the classical alpha rhythm. The work by Sponheim et al. recorded individual alpha-band PFs with MEG during rest and visual processing in participants with psychotic psychopathology, their biological siblings, and healthy controls. Alpha PF varied widely across participants and remained temporally stable across multiple months. Moreover, slowed alpha PF was linked to longer visual percept duration in participants with psychopathology, as well as to lower IQ estimates and higher symptom severity. This study shows how fundamental research can inspire a directed functional assessment of neural markers as indicators of clinical symptom severity.

The systematic and hypothesis-driven analysis of PF holds the potential to provide exciting and novel roles of neural oscillatory activity. It promises to strengthen the analysis of oscillatory power and phase by providing an empirically-informed frame of reference instead of rigid a priori defined frequency definitions. We hope that this Research Topic has increased the visibility of PF in the neuroscientific audience and motivated future research.

## Author contributions

All authors listed have made a substantial, direct, and intellectual contribution to the work and approved it for publication.
